# MiR‐30a‐5p inhibits proliferation and metastasis of hydatidiform mole by regulating B3GNT5 through ERK/AKT pathways

**DOI:** 10.1111/jcmm.15247

**Published:** 2020-06-23

**Authors:** Zhenzhen Guo, Qiannan Sun, Yangyou Liao, Chao Liu, Wenjie Zhao, Xiaoxue Li, Huan Liu, Ming Dong, Yuhong Shang, Linlin Sui, Ying Kong

**Affiliations:** ^1^ Core Lab Glycobiol & Glycoengn College of Basic Medical Sciences Dalian Medical University Dalian China; ^2^ Department of Gynecology First Affiliated Hospital Dalian Med University Dalian China

**Keywords:** miR‐30a, B3GNT5, hydatidiform mole, trophoblast cell

## Abstract

Hydatidiform moles are gestational trophoblastic disease. They are abnormal proliferations of trophoblast cells that have the potential to become cancerous. miR‐miR30a‐5p is a tumour suppressor that participates in the development of numerous diseases. However, the role of miR‐30a in hydatidiform moles and the mechanisms underlying its effects are presently unclear. This study explored the levels of miR‐30a and B3GNT5 expression in human hydatidiform mole tissue. The results showed that miR‐30a and B3GNT5 were differentially expressed in normal placenta and hydatidiform mole, and miR‐30a decreased cell proliferation, invasion and migration in trophoblast cell lines. Upon further examination, it was confirmed that miR‐30a directly targeted the 3’untranslated region of B3GNT5 using a dual‐luciferase assay. The results of the present study also revealed that miR‐30a reduced the proliferation, invasion and migration ability in JAR and BeWo cells by regulating B3GNT5, which may inactivate the ERK and AKT signalling pathways. This study demonstrated that miR‐30a was a novel target B3GNT5 that serves an important role in the development of hydatidiform moles, suggesting that miR‐30a may serve as a novel potential biomarker or useful diagnostic and therapeutic tool for hydatidiform moles in clinical settings.

## 1 INTRODUCTION

A hydatidiform mole (HM), known as a foetal membrane pregnancy, is a subgroup of (gestational trophoblastic diseases) GTDs.^1^ HM is an aberrant human pregnancy characterized by excessive trophoblastic proliferation and abnormal embryonic development. HM has two morphological types, complete (CHM) and partial, and is non‐recurrent. Most available studies on the risk factors of different types of HM and their malignant transformation mainly suffer from the lack of comprehensive genotypic analysis of large cohorts of molar tissues combined with accurate post‐molar hCG follow‐up.^2^ In countries with poor medical healthcare system, moles (mainly CHM) can become invasive and have the increased potential to develop into cancer, especially choriocarcinoma.^3^, ^4^ The risk is higher in HM than in a normal abortion.^5^ HM placentae exhibit a 46,XX or 46,XY androgenic karyotype due to the fertilization of an ovum devoid of DNA by one or two sperms. However, the underlying mechanisms leading to HM‐associated trophoblast hyperplasia remain largely elusive.^6^ Previous research has confirmed that some miRNAs were only expressed in trophoblast cells, such as miR‐517b and miR‐518b which had a markedly low expression in villous stromal cells. This result suggests that these microRNAs are involved in the formation of HM.^7^ miR‐21 was significantly up‐regulated in hydatidiform moles compared with placenta in early pregnancy, and localized to the villus, suggesting that miR‐21 may be the principal cause of the invasive phenotype of GTD.^8^ One demonstrated that the expression of hsa‐mir‐520b, hsa‐mir‐520c and hsa‐mir‐520f were down‐regulated in the peripheral blood of hydatidiform moles and may be candidate microRNAs for the detection of hydatidiform mole in plasma. These microRNAs were located on chromosome 19q13.42 and formed C19MC (the chromosome 19 miRNA cluster).^9^, ^10^

In fact, although most cases of HM may regress after suction evacuation, 8% to 30% of cases of hydatidiform moles may develop into persistent trophoblastic neoplasm that may be affected by some signal pathways.^11^ In recent years, hyperactivation of proto‐oncogenic cell signalling pathways, such as the p21‐activated kinases, leading to aggressive outcome in GTD patients, has been reported.^12^ In addition, GTD is also been characterized by abnormal proliferation and apoptosis^13^, ^14^ and associated with signal pathway regulation. Sekiya found that c‐Rel might play a role in promoting the invasion of choriocarcinoma cells through PI3K/AKT signalling.^15^ miR‐30a‐5p which we studied in this article may play an important role in the regulation of ERK signalling.^16^

B3GNT5 is a member of the beta‐1, 3‐N‐acetylglucosaminyl transferase family and transfers an N‐acetylglucosamine (GlcNAc) from UDP‐GlcNAc to the lactosylceramide for lacto‐neolacto‐chain ganglioside biosynthesis.^17^, ^18^ A methylation array analysis revealed that B3GNT5 was differentially expressed in breast cancer.^19^ Alterations in glycans may be affected and regulated by the expression of B3GNT5, thereby affecting the development of breast cancer subtypes. An additional study reported that B3GNT5 had an evidently lower expression in luminal A and B subtypes, yet a higher expression in basal‐like subtypes.^20^ Encoding tumour‐promoting factors‐B3GNT5 was closely correlated with the expression of COX2 in breast cancer.^21^ The expression of B3GNT5 and TNF‐α was markedly increased in individuals infected with *H pylori*, which demonstrated that the B3GNT5 promoter region included binding sites for the transcription factors NF‐κB and NF‐κB1. The results also demonstrated that TNF‐α activated the NF‐κB pathway and then increased the transcriptional expression of B3GNT5.^22^ It has been hypothesized that miR‐203 may increase the proliferation rate of tumour cells in human hypopharyngeal squamous cell carcinoma potentially via TP63 and B3GNT5.^23^ Kang investigated the development of pre‐ and post‐implantation mice embryos, which had negative effects following treatment with DMSO, and also observed that the expression of B3GNT5 and Wnt3a were decreased in developed blastocysts.^24^, ^25^ Similarly, an additional study observed that B3GNT5, Wnt3a and Eomes were highly expressed following the successful implantation and foetal development.^26^ B3GNT5 is primarily expressed in the spleen, placenta and cerebellar Purkinje cells in adult mice.^18^ Although B3GNT5 is well known to exert a vital function in the progression of cancer and embryo development, the underlying mechanisms of B3GNT5 mediated by miR‐30a remain unclear in hydatidiform moles.

The present study revealed that the level of B3GNT5 was markedly increased in hydatidiform moles when compared with normal placental tissue; however, the expression of miR‐30a was lower. Subsequently, the study identified B3GNT5 as the target of miR‐30a which regulated B3GNT5 and then affected the molecular mechanism governing the development of hydatidiform moles. miR‐30a/ B3GNT5 axis will be an important factor and may be predictive biomarkers in hydatidiform moles.

## 2 MATERIALS AND METHODS

### 2.1 Ethics statements

Twenty formalin‐fixed paraffin‐embedded hydatidiform mole tissues, 10 fresh hydatidiform mole tissues and 15 normal placental tissues were collected from the Dalian Women's and Children's Hospital. The use of these samples and the experimental protocol were approved by the ethics committees of Dalian Medical University. All patients provided written informed consent.

### 2.2 Cell culture

The human choriocarcinoma JAR cell line was obtained from the American Type Culture Collection (ATCC), the BeWo cell line was obtained from the China Center for Type Culture Collection (CCTCC). JAR and BeWo cells were cultured in 90% phenol red‐free RPMI 1640 media (Gibco; Thermo Fisher Scientific, Inc) supplemented with antibiotics (100 U/mL penicillin and 100 μg/mL streptomycin), and 10% foetal calf serum (Gibco; Thermo Fisher Scientific, Inc). All cells were incubated at 37°C in a 5% CO_2_ incubator.

### 2.3 Cell transfection

In order to evaluate the expression level of miR‐30a and B3GNT5, cells at 60% confluency were transfected with miR‐30a mimics/normal control or an anti‐miR‐30a/normal control, which were provided by Guangzhou RiboBio Co., Ltd. The cells were also transfected with B3GNT5 cDNA and B3GNT5 small interfering RNA (siRNA) purchased from Shanghai GenePharma Co., Ltd. The transfection reagent Lipofectamine 2000 (Invitrogen; Thermo Fisher Scientific, Inc) was used according to the manufacturer's protocol.

### 2.4 RNA extraction and reverse transcription‐quantitative polymerase chain reaction (RT‐qPCR)

Total RNA was extracted from the JAR and BeWo cells, using a TRIzol reagent (Takara Biomedical Technology). cDNA was synthesized using a TransScript All‐in‐one First‐Strand cDNA Synthesis SuperMix for qPCR kit (Beijing Transgen Biotech Co., Ltd.). RT‐qPCR analyses were conducted with TransStart Top Green qPCR SuperMix (Beijing Transgen Biotech Co., Ltd) according to the manufacturer's protocol. B3GNT5 (5 ng) was normalized to GAPDH and miR‐30a (20 nmol/L) was normalized to U6, the primer sequences of GAPDH and B3GNT5 were Forward: 5′‐GTGAAGGTCGGAGTCAACG‐3′, Reverse: 5′‐TGAGGTCAATGAAGGGGTC‐3′; Forward: 5′‐ATGCCAAATTCCTGATGACTGT‐3′, Reverse: 5′‐AATTCAGTGTCTGCGATGCCT‐3′. The results were normalized with the 2^−ΔΔCq^ method relative to U6 and GAPDH.

### 2.5 Cell proliferation assay

The cell proliferation of the human choriocarcinoma cells was estimated using the cell counting kit‐8(CCK‐8) (Dojindo Molecular Technologies, Inc) according to the manufacturer's protocol. 5 × 10^3^ cells were seeded in 96‐well plates containing RPMI‐1640 media supplemented with 10% FBS (100 μL) in triplicate for each condition 10 μL CCK‐8 regent was added to each well and the plates were incubated under the aforementioned conditions for 4 hours. Then, the optical density (OD) at 450 nm was obtained using an automatic microplate reader (BioTek Instruments, Inc).

### 2.6 Colony‐formation assay

In order to detect cell proliferation, a colony‐formation assay was performed. A total of 3000 cells per well were seeded in a 6‐well plate and cultured in RPMI‐1640 media supplemented with 10% FBS for ~12 days until the cells grew into a single cell mass that was visible to the naked eye. Cells were then fixed with methanol for 20 minutes and then stained with 0.2% crystal violet for 30 minutes. The number of colonies and cells was then counted.

### 2.7 Migration and invasion assay

We present study performed a transwell assay in order to detect cell migration and invasion abilities. For the migration experiment, 5 × 10^4^ cells were suspended in 200 μL serum‐free medium and added to the upper transwell chamber. Then, an RPMI‐1640 medium containing 10% FBS was added to the lower chamber. Cells were incubated at 37˚C for 24 hours. Following this incubation, the migrated cells were fixed with methanol for 30 minutes and stained with 0.2% crystal violet for 20 minutes. For the invasion assay, the chambers were prepared by pre‐coating with Matrigel (1:20, BD Biosciences) and incubated for 60 minutes 6 × 10^4^ cells were suspended in 200 μL serum‐free medium and added to the upper chamber and 500 μL culture medium containing 10% foetal bovine serum was added to the lower chamber. Following a 36‐hour incubation period, the invading cells were fixed with methanol for 30 minutes and stained with 0.2% crystal violet for an additional 20 minutes. The stained migrated or invaded cells were photographed (×100 magnification), and the cells of five randomly selected fields of view were counted.

### 2.8 Western blot analysis

Whole cell proteins were harvested and the protein concentrations were measured using a BCA kit (Pierce; Thermo Fisher Scientific, Inc). And equal protein (30 μg) was separated with 10% SDS‐PAGE and transferred onto nitrocellulose membranes (EMD Millipore), subsequently, the membranes were then blocked in 5% non‐fat dry milk for 2 hours at room temperature. The membrane was incubated with primary antibodies at 4°C overnight, and the membrane was washed with TBST and incubated with anti‐HPR‐conjugated Affinipure goat anti‐rabbit/mouse lgG(H+L) (1:5000, Proteintech Group, Inc). The signals were detected using an ECL Western blotting kit. The primary antibodies used included: Anti‐B3GNT5 (1:500), anti‐GAPDH (1:3000), anti‐cyclinD1 (1:500), anti‐cyclinB1 (1:500 proteintech), anti‐EGFR (1:1000) (Proteintech Group, Inc) anti‐ERK (1:1000), anti‐p‐ERK (1:500) (Cell Signaling Technology, Inc); AKT (1:1000, Beyotime Institute of Biotechnology) and p‐AKT(Ser473) (1:500, Beyotime Institute of Biotechnology).

### 2.9 Immunohistochemistry and H&E staining

Paraffin sections of hydatidiform mole and normal placenta tissues underwent dewaxing, hydration and antigen repair. Following blocking with serum, the sections were incubated with B3GNT5 antibody (1:70 dilution; Proteintech Group, Inc) at 4°C overnight. The slides were washed with PBS, and incubated with a second antibody at 37˚C for 30 minutes, then streptavidin–horseradish peroxidase was applied at 37˚C for 30 minutes and the slides were stained with 3, 3′diaminobenzidine solution. Images were observed using an inverted microscope. The H&E staining of paraffin‐embedded hydatidiform mole and normal placenta tissues was performed base on the manufacturer's (KeyGen Biotech).

### 2.10 Cell immunofluorescence

Cells were washed three times with cold PBS, fixed with 4% paraformaldehyde for 30 minutes at room temperature, permeabilized in 0.2% Triton X‐100 for 20 minutes at room temperature, and then incubated with 1% BSA for 1 hour at room temperature. Following that, they were stained with the primary antibody‐EGFR(1:70, dilution, Proteintech Group, Inc) overnight at 4°C, and the secondary antibody was applied for 1 hour at room temperature. The cell nucleus was stained with DAPI (Life Technologies; Thermo Fisher Scientific, Inc) and the fluorescence was examined under an Olympus BX51 immunofluorescence microscope (Olympus Corporation).

### 2.11 Dual‐luciferase gene reporter assay

The dual‐luciferase reporter gene experiment was performed using HeLa cells. The wild‐type 3′‐untranslated region (UTR) of B3GNT5 and its mutant were inserted into a pMIR‐RB‐REPORT™ luciferase mRNA expression reporter vector. HeLa cells were seeded at a density of 5 × 10^4^ cells per well in a 6‐well plate transfected using Lipofectamine 2000 (Gibco; Thermo Fisher Scientific, Inc) with the following groups: the miR‐30a mimics or NC mimics (20 nmol/L) with PmiR‐WT‐3’UTR B3GNT5 vector, PmiR‐Mut‐3′UTR B3GNT5 vector. Following a 48 hours transfection, the Firefly and Renilla luciferase activity was assessed using the Dual‐luciferase Reporter Assay System (Promega Corporation) and normalized to the Renilla luciferase activity.

### 2.12 Statistical analysis

Experiments were repeated at least three times. Statistical analyses were performed using GraphPad Prism 7.0 software (GraphPad Software, Inc). These data were presented as the mean ± standard error of the mean. *P* < .05 was considered to indicate a statistically significant difference. Western blots were quantified with ImageJ software and immunohistochemistry and immunofluorescence were quantified using Ipp6.0 software.

## 3 RESULTS

### 3.1 Expression of miR‐30a and B3GNT5 in human hydatidiform moles

**FIGURE 1:**
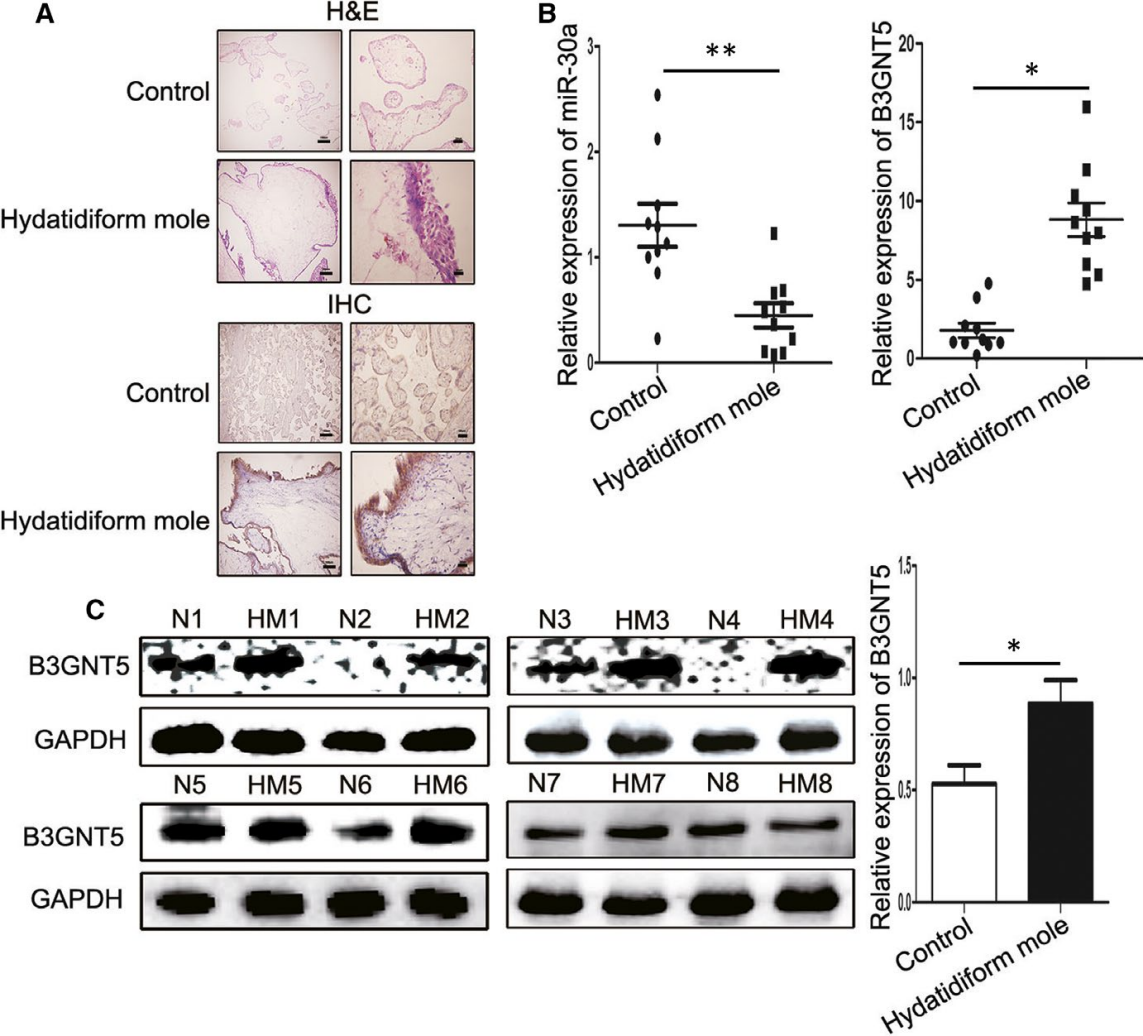
Expression of miR‐30a and B3GNT5 in human hydatidiform moles. (A), Immunohistochemical analysis detected the expression of B3GNT5 in hydatidiform moles and normal placenta, and an H&E staining was performed. (B), RT‐qPCR illustrated the relative expression of miR‐30a and B3GNT5 in hydatidiform mole and normal placental tissues. (C), Western blot analysis for the expression of B3GNT5 in hydatidiform mole tissues and normal placental tissues. GAPDH served as an internal reference. Scale bar = 100 μm, 20 μm, *P < .05, **P < .01 and ***P < .001

Initially, the study identified the expression levels of miR‐30a and B3GNT5 in hydatidiform moles and observed that the expression level of B3GNT5 was dramatically increased in hydatidiform moles compared to normal placental tissue by using IHC‐P (Figure 1A). These results were corroborated by RT‐qPCR, which revealed that the expression level of miR‐30a was lower, and B3GNT5 was higher than normal placental tissue by using RT‐qPCR (Figure 1B). Finally, to test the protein expression of B3GNT5 in the tissue, we performed Western blotting and revealed that although the protein expression of B3GNT5 was decreased in normal placental tissue, its expression was increased in hydatidiform moles (Figure 1C). These results demonstrated that miR‐30a and B3GNT5 had a negative correlation in hydatidiform moles.

### 3.2 Effect of miR‐30a on the proliferation, migration and invasion of trophoblast cells

**FIGURE 2:**
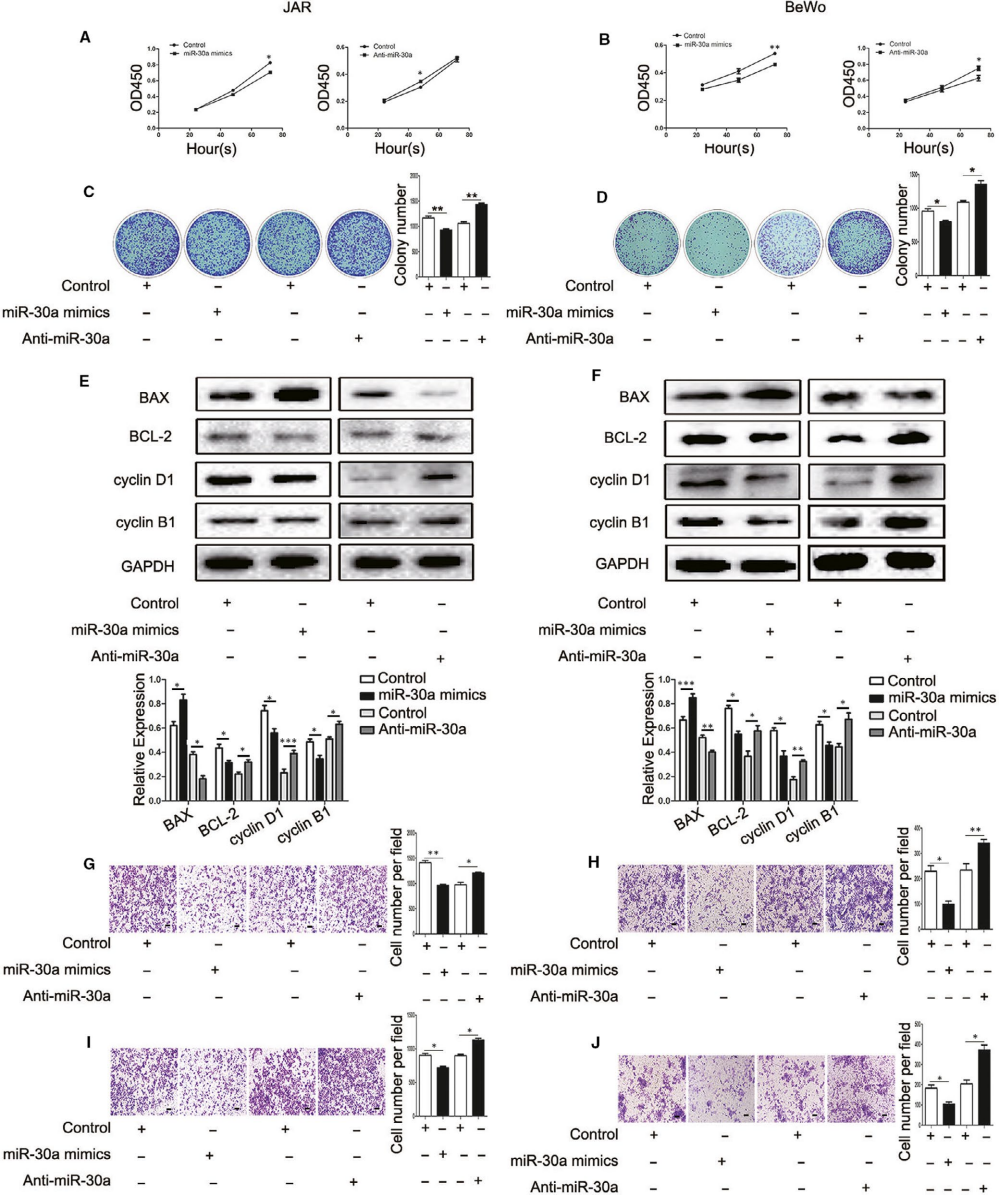
miR‐30a reduced the proliferation, migration and invasion of trophoblast cells. (A and B) The proliferation ability was tested in JAR and BeWo cells transfected with miR‐30a mimics or anti‐miR‐30a by a CCK‐8 assay. (C and D) The capability of cell growth was detected with a colony‐formation assay. (E and F) Western blotting detected the expression levels of cyclin and apoptosis‐associated proteins. (G‐J), A transwell assay was used to evaluate the cell migration and invasion rate of JAR and BeWo cells following transfection with miR‐30a mimics or anti‐miR‐30a. The statistical analysis is shown. Scale bar = 100 μm, *P < .05, **P < .01 and ***P < .001

To verify the mechanism of miR‐30a in the proliferation and metastasis of JAR and BeWo cells, the present study transfected these cells with miR‐30a mimics (miR‐30a) and control or miR‐30a inhibitor (anti‐miR‐30a). The control group was transfected with Lipofectamine 2000. The CCK‐8 assays revealed that the proliferation ability of JAR and BeWo cells was decreased by transfecting with miR‐30a mimics, whereas the proliferation of JAR and BeWo cells was increased following transfection with anti‐miR‐30a (Figure 2A,B). The colony‐formation assay revealed that miR‐30a mimics showed a smaller number of colonies in these cells (Figure 2C,D). Similarly, miR‐30a mimics repressed the expression of cyclin B1, cyclin D1 and Bcl‐2, but increased the expression of BAX as detected by the Western blot assay (Figure 2E,F). We then examined the possible effects of miR‐30a on migration and invasion. The result of the transwell assay demonstrated that the upexpression of miR‐30a led to an attenuated migration rate of JAR and BeWo cells (Figure 2G,H). In addition, the overexpression of miR‐30a markedly reduced the invasive ability of cells (Figure 2I,J). These results showed that the proliferation and metastasis capacity of trophoblast cells were suppressed by transfecting miR‐30a. Further results confirmed that B3GNT5 suppressed the proliferation and metastasis of trophoblast cells (Figure S1).

### 3.3 B3GNT5 was a novel target of miR‐30a

**FIGURE 3:**
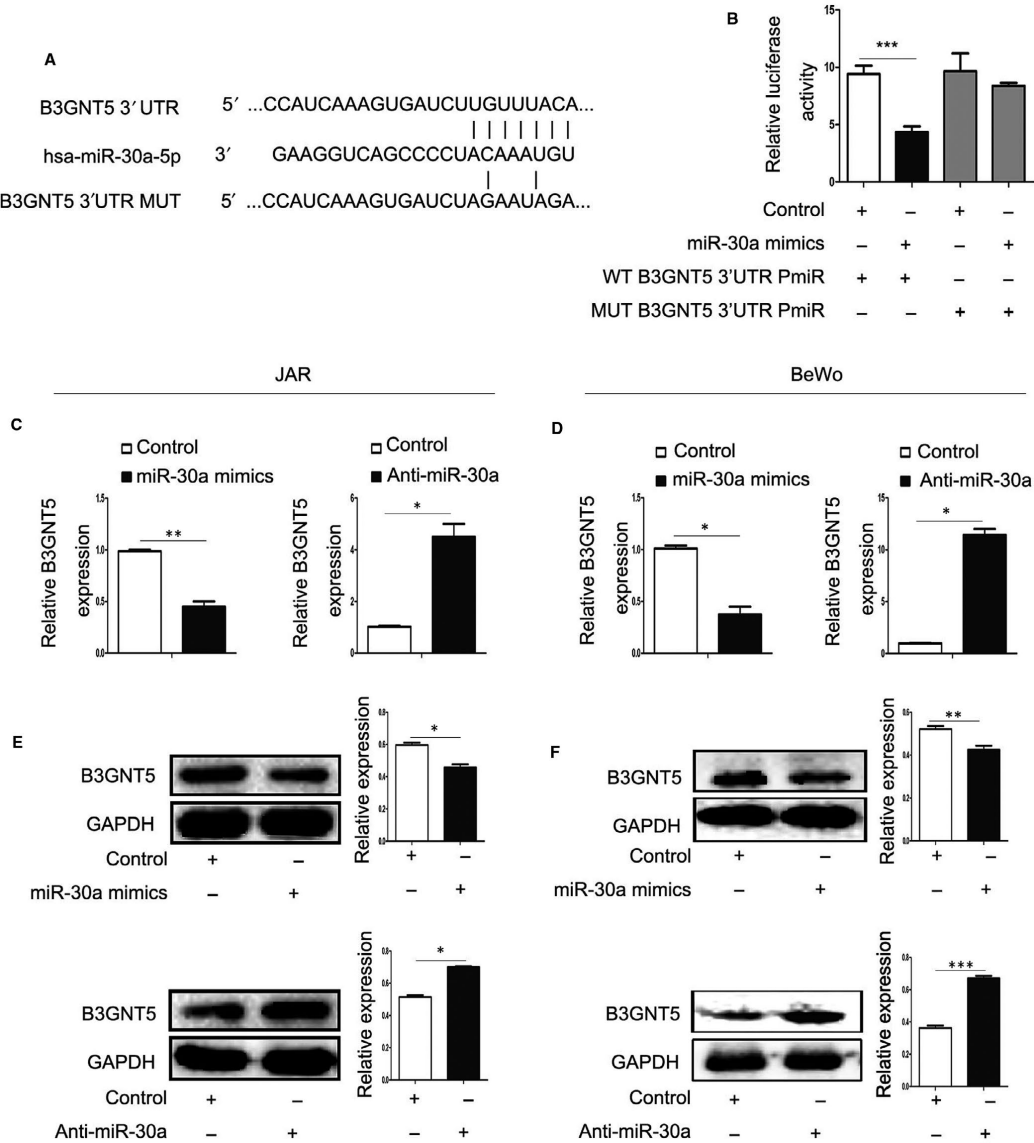
B3GNT5 was a novel target of miR‐30a. (A), The predicted binding sites of miR‐30a and B3GNT5 were portrayed using bioinformatics software, the upper panel represents the reporter constructs of WT‐B3GNT5 3’‐UTR, and the lower panel represents the reporter constructs of Mut‐B3GNT5 3’‐UTR. (B), The target relationship of miR‐30a and B3GNT5 was identified with a dual‐luciferase gene reporter assay in HeLa cells. (C‐F), RT‐qPCR and Western blot assay presented the expression levels of B3GNT5 in JAR and BeWo cells following transfection with miR‐30a mimics or anti‐miR‐30a. *P < .05, **P < .01, ***P < .001

miRNAs have been demonstrated to directly regulate a greater number of target genes in placenta. Therefore, this study predicted the target genes of miR‐30a using the Targetscan, miRanda and miRDB databases. Based on this analysis, B3GNT5 was regarded as a high‐scoring candidate gene for miR‐30a targeting. The results demonstrated that B3GNT5 was a possible target gene and was mapped to the 3’UTR (Figure 3A). In order to verify whether there is a targeting relationship between miR‐30a and B3GNT5, a double‐luciferase reporter gene assay was performed using a reporter construct containing a wild‐type B3GNT5 3’UTR. The luciferase activity was lowly expressed following co‐transfection with miR‐30a mimics in the HeLa cell line; however, there was no alteration with the reporter constructs containing mutant B3GNT5 3’UTR (Figure 3B). Furthermore, when we transfected miR‐30a mimics in JAR cells, the expression of the B3GNT5 protein was lower than the negative control, and B3GNT5 was highly expressed in cells transfected with anti‐miR‐30a (Figure 3C,E). Using these methods, the BeWo cell line was examined, and the same results were obtained (Figure 3D,F). Together, the results suggest that B3GNT5 is a directly target gene of miR‐30a and is regulated by endogenous miR‐30a in trophoblast cell lines.

### 3.4 miR‐30a modulated the proliferation, migration, invasion of trophoblast cells by targeting B3GNT5

**FIGURE 4:**
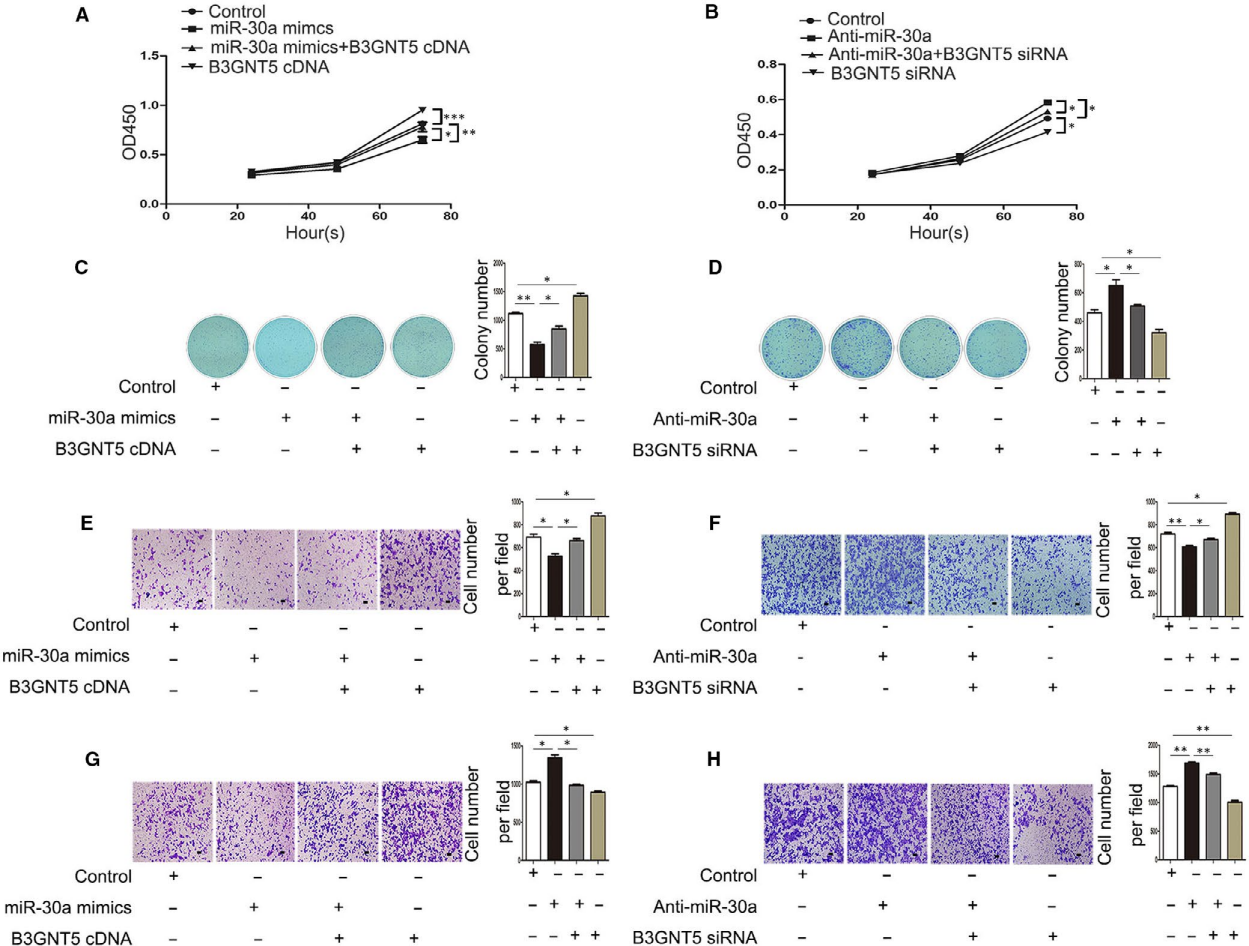
miR‐30a modulated the proliferation, migration and invasion of JAR cells by targeting B3GNT5. (A and B).A CCK‐8 assay and (C and D) colony‐formation assay were used to evaluate the cell proliferation capabilities of JAR cells. (E and F) A transwell assay illustrated the migration rate of JAR cells following transfection with miR‐30a mimics or anti‐miR‐30a. (G and H) Quantification of the transwell results presented the number of invade cells and representative images. Scale bar = 100 μm, *P < .05, **P < .01, ***P < .001

**FIGURE 5:**
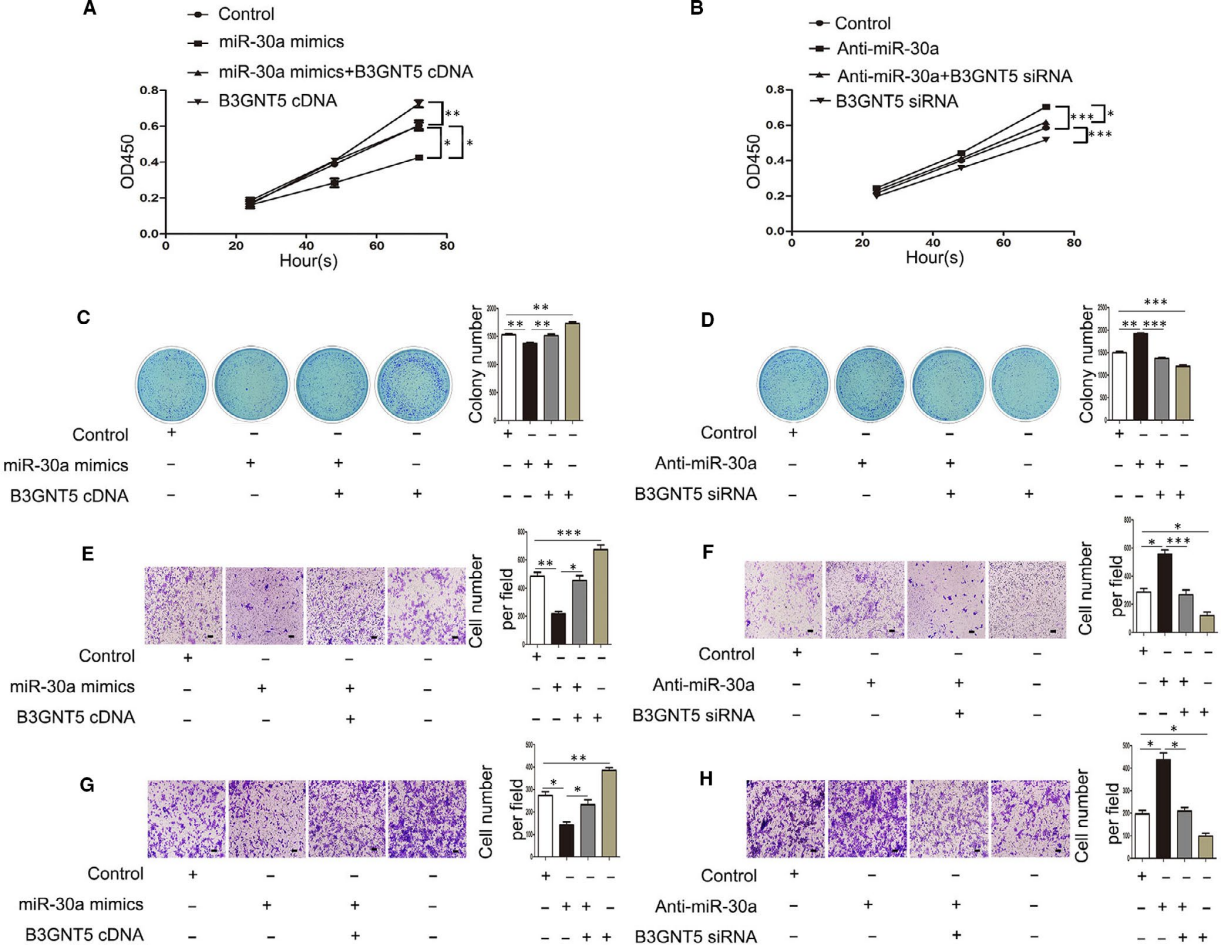
miR‐30a modulated the proliferation, migration, invasion of BeWo cells by targeting B3GNT5. (A and B) The CCK‐8 assay and (C and D) the colony‐formation assay were performed to evaluate the cell proliferation capabilities of BeWo cells. (E and F) The transwell assay demonstrated the migration rate of BeWo cells following transfection miR‐30a mimics or anti‐miR‐30a. (G and H) The results of the transwell assay provided the number of invading cells and representative images are presented. Scale bar = 100 μm, *P < .05, **P < .01, ***P < .001

As described previously, the relationship between miR‐30a and B3GNT5 was demonstrated using a luciferase assay. The study subsequently investigated whether the inhibition of proliferation and metastasis by miR‐30a‐5p is dependent upon B3GNT5 in JAR and BeWo cells. Therefore, a CCK‐8 assay and colony‐formation assay were performed, and the results revealed that increased miR‐30a suppressed the growth rate of JAR and BeWo cells, and the up‐regulation of B3GNT5 boosted the proliferation ability of JAR and BeWo cells; however, following co‐transfection of B3GNT5 cDNA and miR‐30a mimics, the proliferation rate was remarkedly increased compared with transfection of miR‐30a mimics alone. Conversely, B3GNT5 siRNA co‐transfected with anti‐miR‐30a had a lower growth capability than anti‐miR‐30a transfected alone (Figures 4A‐D and 5A‐D). The transwell assay showed similar results and the effect of migration and invasion was confirmed. When we transfected anti‐miR‐30a alone, the migration and invasion of cells were evidently increased, but were decreased following co‐transfection with anti‐miR‐30a and B3GNT5 siRNA in JAR and BeWo cells (Figures 4E‐Hand 5E‐F ). In a word, these results indicated that miR‐30a repressed the proliferation and metastasis of JAR and BeWo cells by regulating the expression of B3GNT5.

### 3.5 miR‐30a mediated ERK, AKT signalling pathways in trophoblast cells via regulating B3GNT5

**FIGURE 6:**
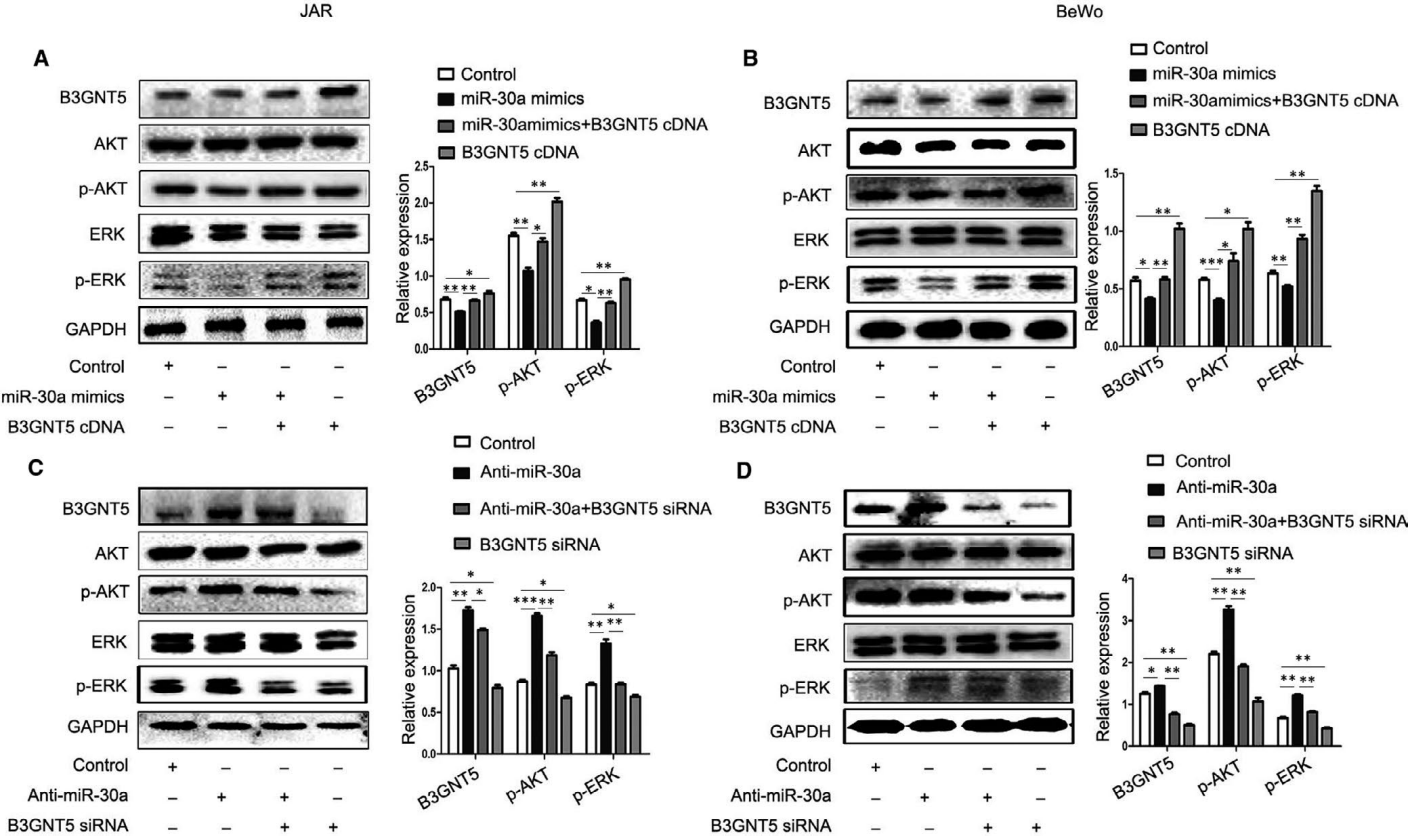
miR‐30a inactivated the ERK/AKT signalling pathways in trophoblast cells via regulating B3GNT5. (A and B) Western blot analysis of the phosphorylation levels of ERK and AKT when cells were transfected with miR‐30a mimics or co‐transfected with miR‐30a mimics and B3GNT5 cDNA or B3GNT5 cDNA, respectively. (C and D) Western blot analysed the phosphorylation level of ERK and AKT when transfected with miR‐30a inhibitor or co‐transfected with miR‐30a inhibitor and B3GNT5 siRNA or B3GNT5 siRNA, respectively. *P < .05, **P < .01 and ***P < .001

To clarify the molecular mechanisms underlying the functional role of miR‐30a and B3GNT5, the study demonstrated that miR‐30a could activate the ERK and AKT pathways by targeting a diverse range of genes.^27^, ^28^ In order to gain further insight into whether miR‐30a activates the ERK and AKT pathways by regulating the expression of B3GNT5, Western blotting was performed by transfecting miR‐30a mimics and B3GNT5 cDNA or B3GNT5 cDNA alone, into the examined cell lines. The results demonstrated that the expression of the B3GNT5 protein was up‐regulated in the co‐transfected miR‐30a mimics and the B3GNT5 cDNA group compared with that in the miR‐30a mimics group alone, subsequently, showing the effect of miR‐30a mimics and B3GNT5 cDNA on activation of the ERK, AKT signalling pathways. The expression of ERK and AKT protein were not changed, yet the expression of p‐ERK and p‐AKT protein were obviously lower in the co‐transfected miR‐30a mimics and B3GNT5 cDNA group than the B3GNT5 cDNA group (Figure 6A,B). It is of note that p‐ERK and p‐AKT proteins were increased following co‐transfection with anti‐miR‐30a and B3GNT5 siRNA, more so than with B3GNT5 siRNA alone, yet the total amount of ERK and AKT proteins remained unchanged (Figure 6C,D). In summary, these data confirmed that overexpressed miR‐30a reduced the activation of the ERK and AKT pathways by regulating B3GNT5.

### 3.6 miR‐30a and B3GNT5 affects the expression of epidermal growth factor receptor (EGFR)

**FIGURE 7:**
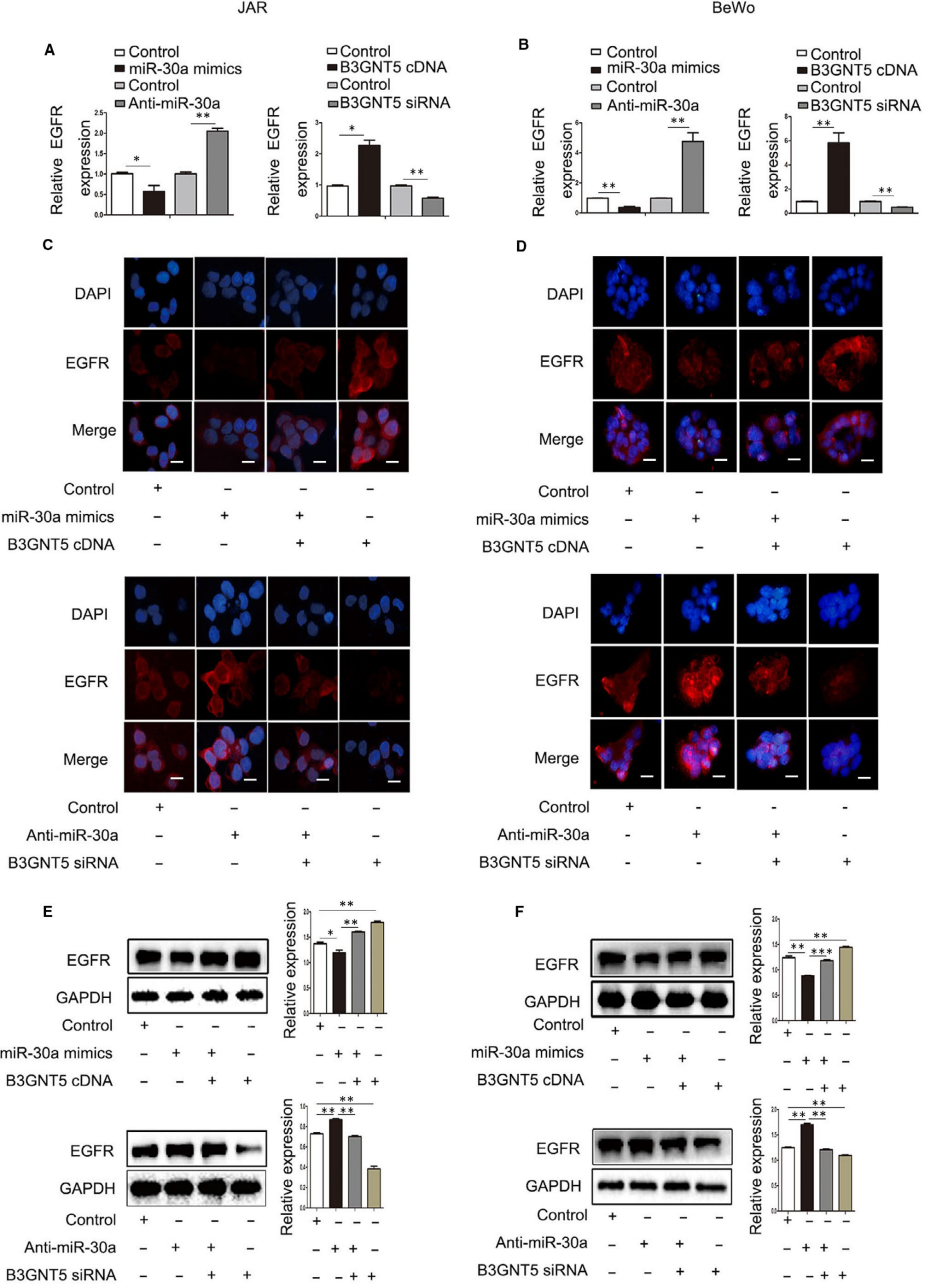
miR‐30a and B3GNT5 affects the expression of EGFR. (A and B) The mRNA expression of EGFR was analysed using RT‐qPCR when JAR and BeWo cells were transfected with miR‐30a mimics or anti‐miR‐30a. (C and D) Immunofluorescent staining of B3GNT5 was observed following transfection with miR‐30a mimics or anti‐miR‐30a in JAR and BeWo cells. (E and F) The expression of the EGFR protein was detected following transfection with miR‐30a mimics or co‐transfection with miR‐30a mimics and B3GNT5 cDNA or B3GNT5 cDNA, respectively. Scale bar = 20 μm, *P < .05, **P < .01 and ***P < .001

EGFR is a transmembrane glycoprotein that is associated with numerous biological processes such as cell proliferation, apoptosis, angiogenesis and metastasis. Previous studies have reported that EGFR is correlated with the development of hydatidiform moles and is also highly expressed in hydatidiform moles.^29^, ^30^ The results indicated that miR‐30a could exert pivotal function in such moles by targeting B3GNT5. Thus, the present study attempted to establish whether the expression of EGFR was regulated by miR‐30a and B3GNT5. qPCR results revealed that EGFR exerted a downward expression trend following transfection with miR‐30a mimics or B3GNT5 siRNA, and also revealed that the expression of EGFR was increased in the anti‐miR‐30a group or the B3GNT5 cDNA group (Figure 7A,B). When we co‐transfected miR‐30a mimics and B3GNT5 cDNA in JAR and BeWo cells, immunofluorescent staining of EGFR demonstrated that the fluorescence intensity was attenuated compared with that of cells transfected with B3GNT5 cDNA and augmented compared with that of cells transfected with miR‐30a mimics only (Figure 7C,D). The Western blotting EGFR results exhibited similar alterations (Figure 7E,F). The data indicated that miR‐30a and B3GNT5 regulate the expression of EGFR by altering Le^x^ biosynthesis.

## 4 DISCUSSION

Hydatidiform mole is an abnormal placental villus oedema and trophoblast proliferation that has a high potential to develop into a gestational trophoblastic neoplasia.^31^ The abnormal expression of miRNAs has previously been reported in a diverse range of diseases, including hydatidiform moles, and miRNAs may function either as oncogenes or anti‐oncogenes. For example, Miura *et al* studied the clinical application of microRNAs associated with hydatidiform moles, including hsa‐miR‐520b, hsa‐miR‐520f and hsa‐miR‐520c‐3p, and revealed that the trend of miRNAs in plasma was consistent with that of the hCG concentration in serum, which indicated that microRNAs may be used as biomarkers for the clinical detection of hydatidiform moles.^32^ Another previous study reported that the miRNA‐21 level was markedly elevated in hydatidiform mole tissues compared with normal placental tissues and that miRNA‐21 was primarily confined to the trophoblastic layers.^8^ Therefore, our results indicated that miR‐30a‐5p exerts a repressible effect on hydatidiform moles. In addition, Wei showed that miR‐30a suppressed the progression of lung cancer via regulating the p38 MAPK pathway by targeting DNMT3A in lung cancer.^33^ This suggests that miRNA may be a perspective in the study of hydatidiform moles and may provide a novel and specific index for its detection in a clinical setting.

Although the members of the miR‐30 family have a common conserved seed sequence, their regulatory mechanisms are executed differently, due to differences in the targeting genes. Metadherin is a transmembrane protein, and up‐regulated miR‐30a has been observed to reduce the expression of metadherin, increase the expression of PTEN and inhibit the phosphorylation of AKT, which ultimately represses the proliferation rate of cells.^34^ It has been reported that miR‐30a could arrest cell cycle progression at the G0/G1 and G1/S phases, which is accompanied by the induction of apoptosis by activating different biological pathways.^35^, ^36^, ^37^ A study by Zhong et al demonstrated that miR‐30a repressed the invasion and migration of colorectal carcinoma through inhibiting the PI3K/AKT/mTOR pathways via directly targeting PIK3CD.^27^ Using a microarray analysis, a previous study detected that the expression of miR‐30a was higher in mesenchymal stem cells from pregnant women with severe preeclampsia (sPE dMSCs) than in normal pregnant women. This may be due to the inhibition of IL‐1β, and the subsequent activation of the NF‐κB and JNK signalling pathways by reducing the expression of TAB3, indicating miR‐30a serves an important role in the immune dysregulation of the maternal‐foetal interface in MSCs during PE.^38^, ^39^ However, the exact relationship miR‐30a and B3GNT5 in hydatidiform mole has yet to be completely elucidated. The B3GNT5 gene encodes the glycosyltransferase β‐1,3‐N‐acetylglucosaminyl transferase 5, which attaches N‐acetylglucosamine (GlcNAc) to lactosylceramide (Galβ1‐4Glcβ1‐1Ceramide) resulting in the precursor lactotriaosylceramide (Lc3, GlcNAcβ1‐3Galβ1‐4Glcβ1‐1Ceramide) for synthesis of lacto and neolacto‐series GSLs.^17^ This enzyme, together with its associated glycosidic product (Lc3), plays a role in human malignant diseases, embryonic development and cell differentiation.^40^ Specifically, Lc3 was shown to be elevated on the cell surface of human pro‐myelocytic leukaemia HL60 cells.^41^ Additionally, the expression of B3GNT5 was revealed to be markedly decreased in the high‐grade ovarian cancer cell line A2780; however, it was increased in its cisplatin‐resistant subline A2780‐cp.^42^ B3GNT5 has also been identified as a tumour‐promoting gene.^21^ Our results suggest that miR‐30a may affect the development of HM by directly regulating B3GNT5.

A previous study established that miR‐30a was involved in hydatidiform moles via targeting B3GNT5; however, the biological mechanisms governing this interaction are presently unclear. The ERK and AKT signalling pathways are closely associated with human diseases and participate in the cell proliferation, differentiation, metastasis and metabolism of various biological functions.^43^, ^44^ In recent years, numerous reports have attested to the fact that miRNAs play an important role in the development of various diseases by regulating multiple signalling pathways through different target genes. A previous study reported that miR‐30a reduced the proliferation ability via regulating p‐AKT and P53 by targeting IGF1R in melanoma cells.^45^ In addition, Fu *et al* demonstrated that the overexpression of miR30a could inhibit the expression of Neurod 1, which then prevented inflammatory responses and oxidative responses through the MAPK/ERK signalling pathway.^46^ In the present study, miR‐30a mimics or B3GNT5 siRNA transfection decreased the expression levels of p‐ERK and p‐AKT; however, the amount of ERK and AKT was unchanged in the JAR and BeWo cell lines. Conversely, miR‐30a and B3GNT5 were up‐regulated and activated the ERK and AKT pathways.

EGFR is the receptor of tyrosine kinase (RTK). It consists of C‐terminal intracellular domain with kinase activity and N‐terminal extracellular ligand binding site with hydrophobic transmembrane domain.^47^ After binding with ligand, it can activate many downstream pathways, including Ras/ Raf/ mitogen‐activated protein kinase (MAPK) axis, which is mainly involved in cell proliferation and inositol phosphate, and (PI3K)/ PTEN/ Akt axis, which is involved in cell activity and movement.^48^ EGFR is regulated by the surrounding lipid environment. In addition to the more general effect of lipids (such as cholesterol) on the biophysical properties of membrane, this regulation may occur through specific lipid interactions.^49^ Among them, glycosphingolipids have been shown to regulate EGFR activity.^50^ The EGFR is closely involved in the proliferation and metastasis of cell and serves as a primary therapeutic target.^51^, ^52^ It has been reported that the pY291‐Fas promotes the nuclear localization of p‐EGFR and p‐STAT3, and the expression of cyclinD1 through the AKT and MAPK pathways.^53^ EGFR, VEGR, and c‐jun have been observed to be expressed in placental trophoblasts during different stages of gestation and served an important role in trophoblast proliferation metastasis and metabolic activity.^54^ Hydatidiform moles are caused by the excessive proliferation of trophoblast cells, which are epithelial in origin; however, it is unclear whether EGFR is involved in the pathogenesis of HM.^55^, ^56^ The results of our study demonstrated that EGFR was affected by miR‐30a, and regulated the expression of B3GNT5 in JAR and BeWo cells. However further research is required in order to completely elucidate the molecular mechanism by which miR‐30a and B3GNT5 affect EGFR.

In summary, to the best of our knowledge, the present study is the first to demonstrate that the expression level of miR‐30a is lower in hydatidiform mole compared with that in normal placenta. In addition, this study also emphasized the existence of a novel relationship between miR‐30a and B3GNT5, which may impact the inhibition of the proliferation, migration and invasion of trophoblast cells, and further inactivate the ERK and AKT signalling pathways. In short, the present study provided a novel strategy for the diagnosis of hydatidiform mole in a clinical setting.

## CONFLICT OF INTEREST

The authors declare they have no competing interests.

## AUTHOR CONTRIBUTION

Zhenzhen Guo and Qiannan Sun performed experiments; Yangyou Liao analysed data; Chao Liu, Wenjie Zhao were responsible for cell culture; Xiaoxue Li, Huan Liu organized the analytical laboratory and provided experimental advice; Yuhong Shang collected endometrial tissues; Ming Dong generated the figures and wrote the manuscript. Linlin Sui and Ying Kong conceived and organized the study, analysed data. All authors contributed to drafting or editing of the manuscript.

## Supporting information

Fig S1

## Data Availability

All data generated or analysed during this study are included in this article.
