# Supplementary figures and images for: MiR‐30a‐5p inhibits proliferation and metastasis of hydatidiform mole by regulating B3GNT5 through ERK/AKT pathways
Source: J Cell Mol Med. 2020 Jun 23;24(15):8350–62. doi: 10.1111/jcmm.15247 (PMC7412694; doi:10.1111/jcmm.15247)

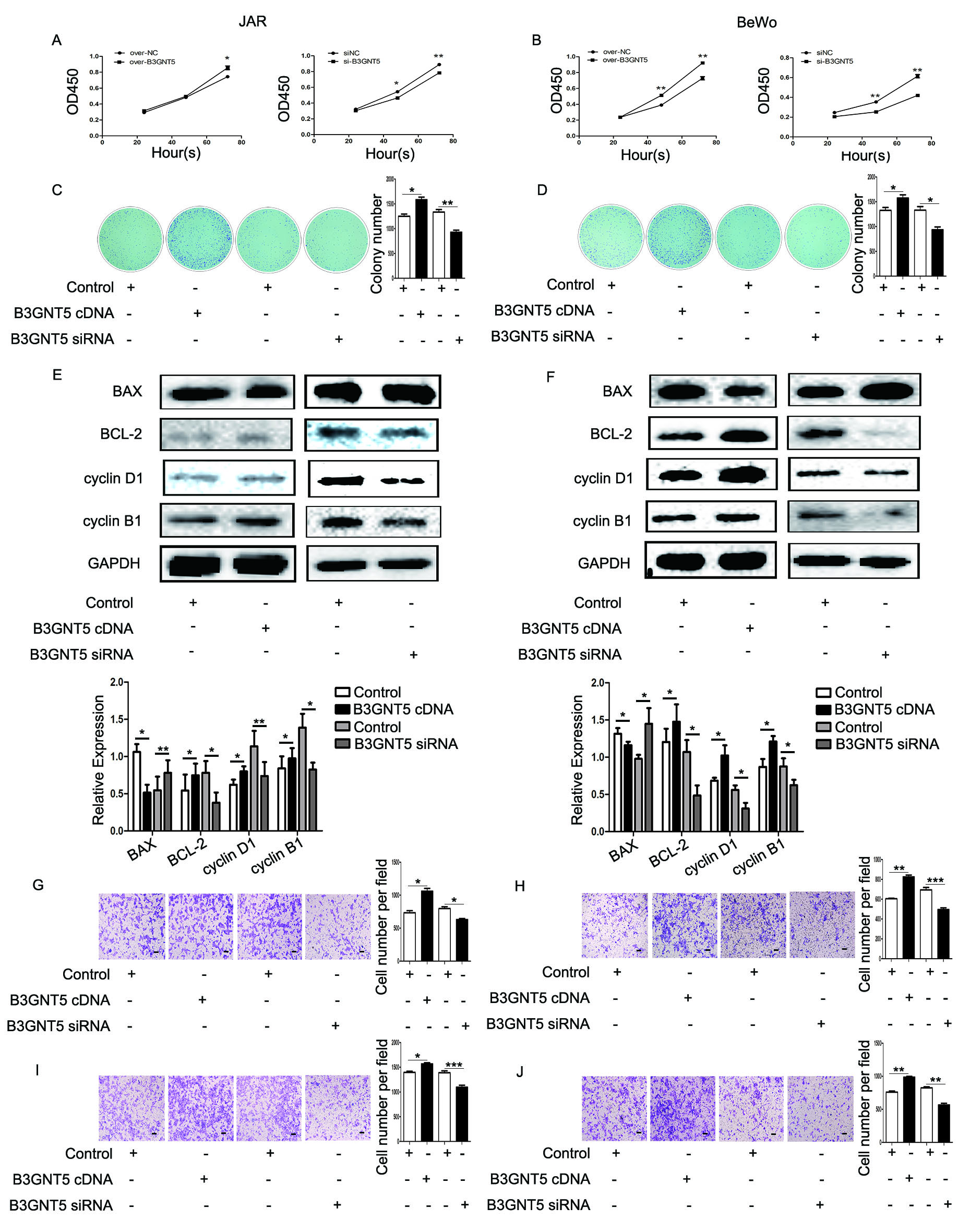

Supplement: Supplementary file 1 — Fig S1 [file JCMM-24-8350-s001.jpg]
